# Supplementary material for: Verification of Hypsibius exemplaris Gąsiorek et al., 2018 (Eutardigrada; Hypsibiidae) application in anhydrobiosis research
Source: PLoS One. 2022 Mar 18;17(3):e0261485. doi: 10.1371/journal.pone.0261485 (PMC8932574; doi:10.1371/journal.pone.0261485)
Supplement: Attachment — Submitted filename: Propawa-et-al-Review-2021-1.docx [file pone.0261485.s001.docx]

General comments:

In this manuscript, Propawa et al. used the tardigrade *Hypsibius exemplaris* species and three dehydration protocols to asses for the shape of the formed tuns, the ultrastructure of the storage cells as well as survival during rehydration.

The anhydrobiosis protocols used in this study showed variable efficiency in the formation of viable tun when assessed for rehydration after 7 days. Moreover, with the use of the transmission electron microscopy for ultrastructural analysis of the anydrobiotic specimens, the authors describe three storage cells degeneration status that can be correlated with a gradual failure of tuns formation depending on the anhydrobiosis protocol used.

The authors then concludes that only tuns without visible degeneration of storage cells are able to support a successful rehydration process.

This manuscript needs major revisions which concern the quality of the images presented and the final conclusions drawn from them.

Specific issues:

1. Line 84: There is no precise description of the experimental “environmental drying” protocol. Why were these two types of substrates chosen?

Please clarify these two points.

2. Line 96: Why the described protocol uses "double-distilled water and Spring water" instead of the classical "Chalkley" medium (<http://cshprotocols.cshlp.org/content/2018/11/pdb.prot102319.full>)  ? Please specify.
3. Line 108: The anhydrobiosis protocol refers to the following work (<http://cshprotocols.cshlp.org/content/2018/11/pdb.prot102327.full.pdf>) in which tardigrades are starved a day before the dehydration operation. Do the specimens also starved as described in the cited reference? Please specify.
4. In line 125, the authors have described the substrate C1 and in line 128 they describe a substrate consisting of soil and decomposed plants. Same remark as above (1): Why were these two types of substrates chosen? Please clarify.
5. Figure 1: The two images of tuns are totally different and cannot be compared to each other. As stated in the material and methods section, they were taken with different stereomicroscopes without using an identical exposure. The right-hand picture does not clearly distinguish the specimen. The resolution of the images is sub-standard. No scale bar is shown. Please present better quality images.
6. The fixation protocol with 2.5% glutaraldehyde is repeated twice on lines 148 and 151.
7. lines 150 to 153 describe two completely different protocols A and B, whose observations were made with different stereomicroscopes (Olympus SZ61 and Olympus BX60). As mentioned above (5), it seems difficult to compare both specimens. The conclusions drawn from these observations must be modified accordingly.
8. To perform TEM analysis, it is indicated in line 131 that 5 specimens are used while in line 153 it is indicated that 10 specimens are analyzed. Are there 10 specimens in total that were analyzed in TEM or 10 specimens per experimental condition? Please clarify.
9. The lines 175-177 describe observations based on Figure 1. As noted above (5 and 7), these figures are not of good enough quality to compare the two types of anhydrobiosis protocols. In addition, the observation that the appearance of the tun in Figure 1 in the lower right is good is impossible to verify because of the poor quality of the stereomicroscopy image. A change to Figure 1 with better quality stereomicrocopy images is required.
10. Line 181: identical question like in (8) above. Were 10 individuals analyzed for A and 10 individuals for B or were only 5 individuals analyzed? Please clarify.
11. Line 186: TEM ultrastructural analysis of specimen integrity is based on a previous study that involved only storage cells from another tardigrade species. Does the analysis of a single cell type provide sufficient information to perform the classification of degenerative states proposed in this manuscript?
12. Line 192: A description of the mitochondria is made based on Figure 3A. However, the quality of the image does not allow to observe the state of the mitochondria or to detect the presence of mitochondrial cristae? The quality of Figure 3A needs to be improved to be interpreted with certainty.
13. Line 194: a description of Figure 3B shows "single vacuole and autophagosomes appeared". However, in Figure 3C corresponding to stage 0 a vacuole also appears.
14. Line 198: A description of the mitochondria in Figure 3C is presented. However, the quality of the images does not allow for the degree of interpretation that is proposed. A better image quality is required to allow support for the proposed observations.
15. Lines 202 and 203: it is stated that "these observations corroborated with the survival rate" However, while the observations indicate 100% of storage cells degeneration one can also note that the corresponding mortality which is not 100% maintains a low survival rate. The analysis of only the storage cell type may not be discriminating enough to allow a totally reliable prediction in the absence of the analysis of at least one other cell type. Please adjust the conclusion of this section accordingly.
16. Line 212: "we ca assume.." should be corrected to ""we can assume.."

Figure caption:

Figure 1: Missing figure description information of the tardigrades tun in the center of the figure.

Figure 2: It is missing the description of the statistical t-test which is present in the material and methods section but absent from the figure legend.

Figure 3: The scale bars are very different: 0.38 for A and 0.53 for B. It is necessary to modify the images so that the scales are homogeneous and in correspondence.
