# Supplementary material for: Verification of Hypsibius exemplaris Gąsiorek et al., 2018 (Eutardigrada; Hypsibiidae) application in anhydrobiosis research
Source: PLoS One. 2022 Mar 18;17(3):e0261485. doi: 10.1371/journal.pone.0261485 (PMC8932574; doi:10.1371/journal.pone.0261485)
Supplement: Attachment — Submitted filename: Responses to reviewers.docx [file pone.0261485.s002.docx]

*Dear Editor,*

*Thank you very much for the constructive reviews and the positive comments on our manuscript. We have addressed all comments made by the Reviewers and marked our answers (in red) by ‘REPLY’. We also send you the corrected manuscript in two versions: one with all changes made visible (‘Revised Manuscript with Track Changes') and the other with all changes merged with the text (‘Manuscript’).*

*Kind Regards,*

**Editor**

Dear Authors, two external reviewers have now assessed your manuscript "*Verification of Hypsibius exemplaris Gąsiorek et al., 2018 (Eutardigrada; Hypsibiidae) application in anhydrobiosis research*”, providing the comments that are reported below. As you can see, they both found identified a number of issues that would require careful revision before this paper is recommendable for acceptance.

Based on the reviewers' assessment, I'm thus here inviting you to take all of these comments into careful consideration and to modify your manuscript according to the provided constructive suggestions. I will then be happy to receive and further examine your revised version together with a point-by-point reply to each comment by myself and each reviewer, where you will need to explain any changes done to a particular piece of text, or include supported and convincing counterarguments to any points you may disagree with I'm confident you will find the present comments and suggestions relevant and useful to improve your work and I'm thus looking forward to hearing back form you by the due time.

REPLY: Dear Editor, thank you for your work on our manuscript. We considered all suggestions made by Reviewers and accepted all of them.

Please include the following items when submitting your revised manuscript:

- A rebuttal letter that responds to each point raised by the academic editor and reviewer(s). You should upload this letter as a separate file labeled 'Response to Reviewers'.
- A marked-up copy of your manuscript that highlights changes made to the original version. You should upload this as a separate file labeled 'Revised Manuscript with Track Changes'.
- An unmarked version of your revised paper without tracked changes. You should upload this as a separate file labeled 'Manuscript'.

REPLY: Prepared as indicated.

Journal Requirements:

When submitting your revision, we need you to address these additional requirements.

1. Please ensure that your manuscript meets PLOS ONE's style requirements, including those for file naming. The PLOS ONE style templates can be found at https://journals.plos.org/plosone/s/file?id=wjVg/PLOSOne_formatting_sample_main_body.pdf and https://journals.plos.org/plosone/s/file?id=ba62/PLOSOne_formatting_sample_title_authors_affiliations.pdf.

REPLY: Checked and corrected as indicated.

2. Thank you for stating the following in the Acknowledgments Section of your manuscript: 
[These studies were supported by the research grant of National Science Centre, Poland, NCN 2016/21/B/NZ4/00131. The technical contribution of Kamil Janelt is highly appreciated.
Studies have been conducted in the framework of activities of BARg (Biodiversity and Astrobiology Research group).]

We note that you have provided funding information that is not currently declared in your Funding Statement. However, funding information should not appear in the Acknowledgments section or other areas of your manuscript. We will only publish funding information present in the Funding Statement section of the online submission form. 
Please remove any funding-related text from the manuscript and let us know how you would like to update your Funding Statement. Currently, your Funding Statement reads as follows: 
 [These studies were supported by the research grant of National Science Centre, Poland, NCN 2016/21/B/NZ4/00131.]

Please include your amended statements within your cover letter; we will change the online submission form on your behalf.

REPLY: Corrected as indicated.

**Reviewer 1**

This manuscript describes several alternative ways to dehydrate tardigrades of the species *Hypsibius exemplaris*. The alternative means used are interesting but the manuscript lacks precision in the motivation of these experimental choices. The quality of the images presented does not fully support the conclusions presented in the manuscript and needs to be improved.

REPLY: We tried to increase our precision in explaining the motivation behind our experimental choices by adding the relevant explanation to the manuscript (at the end of the Introduction section). We also improved the quality of images as suggested by the Reviewer.

**Reviewer 2**

General comments: In this manuscript, authors used the tardigrade Hypsibius exemplaris and three dehydration protocols to study the shape of the tuns, the ultrastructure of the storage cells and the survival during rehydration.

After seven days, the three anhydrobiosis protocols used in this study showed variable efficiency in the formation of viable tun. The transmission electron microscopy analysis of tuns specimens showed storage cells degeneration status. Those status can be correlated with variable efficiency in the formation of viable tun. Propawa et al. concluded that only tuns without visible degeneration of storage cells are able to support a successful rehydration process.

This manuscript needs major revisions. Indeed, the poor quality of the pictures doesn’t allow to conclude anything.

REPLY: Thank you for these comments. We corrected our manuscript according to the comments and also improved the image quality.

**Specific comments:**

*Text:*

1. Line 84: There is no reference or accurate description of the experimental protocol for “environmental drying”. Why did authors choose these two substrates?

REPLY: The Reviewer is right. Therefore, we added the following sentence (with proper references): “Fine sand and pond sediments are natural habitats for freshwater tardigrades including *Hys. exemplaris*, which motivated our decision to use of them as inorganic and organic drying substrates respectively”. The protocol and the substrates are described in more detail in the section Materials and Methods: Anhydrobiosis protocols.

2. Line 96: Propawa et al. used "double-distilled water and Spring water" instead of the classical Chalkley medium (http://cshprotocols.cshlp.org/content/2018/11/pdb.prot102319.full). Please justify this choice.

REPLY: The analysis of the available culturing methods of *Hys. exemplaris*, including the applied media, indicated that the classic Chalkley medium is commonly replaced by spring water mixed with distilled water in different ratio (for review, see Roszkowska et al. (2021) Tips and tricks how to culture water bears: simple protocols for culturing eutardigrades (Tardigrada) under laboratory conditions. Eur Zool J.88(1): 449–465; doi:10.1080/24750263.2021.1881631). It appears that the classic Chalkley medium is better for large scale culture performed in Erlenmeyer flasks whereas small scale cultures performed in different kind of plates, are performed in the mix of spring water and distilled water. Accordingly, the application of spring water is also mentioned in the indicated link to the protocol by Robert McNuff. As our experiments consisted of analysis of anhydrobiosis survival we needed a small scale culture to control the survival, so we applied Petri dishes and the indicated mix of spring water and distilled water, and this approach is often used by different tardigrade groups, including our laboratory.

3. Line 108: In this work, authors mentioned Boothby’s protocol (http://cshprotocols.cshlp.org/content/2018/11/pdb.prot102327.full.pdf). In this protocol, tardigrades are starved a day before dehydration. In the present work, it’s not described? Please specify if a starvation was conducted.

REPLY: We are sorry for the omission. The relevant information was added.

4. In line 125, the authors have described the substrate C1 and in line 128 they describe a substrate consisting of soil and decomposed plants. Same remark as above (1): Why were these two types of substrates chosen? Please clarify.

REPLY: The Reviewer is right. The answer to the Reviewer’s question is given at the end of the Introduction section (see also the answer to the comment 1).

5. Line 148 + Line 151: Authors mentioned twice the same fixation protocol with 2.5% glutaraldehyde. Only one is enough.

REPLY: We are sorry for the repetition. This part was changed in the revised version of the manuscript because we decided to use the same stereomicroscope to make images of tuns obtained by protocols A and B as well as the same background for the images. This part was changed as follows:

“Randomly selected representative tuns were photographed on agar plates using the stereomicroscope. Tuns obtained by protocol B before transferring to agar plates were fixed in solution of 2.5% glutaraldehyde in 0.1 M sodium phosphate buffer (pH 7.4) at room temperature for 2 minutes. The fixation of the tuns was necessary to extract them from the filter paper fibres. Tardigrades penetrate the filter paper fibres during tun formation and the tuns cannot be removed without the filter paper being wet. Thus, the use of a fixative prevents rehydration of specimens and allows removal of the tuns from the dried filter paper.“

6. Line 150 to Line 153: Protocols A and B are totally different. Tuns obtained by protocol A were photographed on agar plates using an Olympus SZ61 stereomicroscope. Tuns obtained using protocol B were fixed in 2.5% glutaraldehyde in 0.1 M sodium phosphate buffer, mounted in a drop of water on a slide, covered by coverslip, and photographed with the use of Olympus BX60 stereomicroscope and OLYMPUS DP50 camera. It is impossible to compare both specimens. The conclusions drawn from these observations must be modified accordingly.

REPLY: As we wrote in the answer to the comment 5, we changed the procedure of preparing images of tuns obtained by protocol B, i.e. the tuns were photographed on agar plates and using the same stereomicroscope as in the case of tuns obtained by protocol A. We hope that after this change both types of tuns are comparable. We cannot avoid the fixation of tuns obtained by protocol B because such tuns penetrate the filter paper fibres and cannot be removed without the filter paper being wet. The use of a fixative prevents rehydration of specimens.

7. Line 154: Authors indicated that 10 specimens were analyzed. Is it 10 specimens in total or 10 specimens per experimental condition? It is not clear. Indeed, line 131, 5 specimens are mentioned.

REPLY: Ten tuns were randomly selected for each of the protocols. To improve the sentence, we changed it as follows: “Out of tuns obtained by protocols A and B, 10 tuns were randomly selected for each of the protocols to perform ultrastructure analysis under transmission electron microscopy (TEM).”. We also changed Table 2 caption relevantly and after the change it is as follows: **“**Summary of ultrastructural analysis of storage cells in typical tuns. For the analysis, 10 tuns obtained by protocol A and 10 tuns obtained by protocol B were randomly selected.”.

As far as the comment “Indeed, line 131, 5 specimens are mentioned” is concerned:.

Five additional specimens were added to each plate to have the proper amount of tuns for survival analysis (as indicated in Table 1) as well as for tuns’ photography under a stereomicroscope and TEM analysis (in the case of the latter, 10 randomly selected for each of the mentioned protocols). To improve the clarity of the approach, we changed the sentence as follows: “In the case of the A and B protocols, each plate contained five additional specimens for tuns’ photography under stereomicroscope and tuns’ ultrastructure analysis under transmission electron microscopy (see below)”. We also added the information to caption of Table 1.

8. Lines 175 to 177 describe observations based on Figure 1. As noted above (5 and 7), these figures are not of good enough quality to compare the two types of anhydrobiosis protocols. In addition, the observation that the appearance of the tun in Figure 1 in the lower right is good is impossible to verify because of the poor quality of the stereomicroscopy image. A change to Figure 1 with better quality stereomicrocopy images is required.

REPLY: As stated in the answers to the comments 5 and 6, we changed the procedure of preparing images of tuns obtained by protocol B and corrected the Figure accordingly. We hope that tuns in the corrected images are now comparable. Figure 1 shows the research scheme. Therefore, images obtained by TEM are small and have no scale bars. Figure 3 contains the bars as well as images of much better quality. We added the relevant information to the caption of Figure 1. The information is as follows: “High definition images obtained using TEM analysis, with relevant scale bars added, are presented in Figure 3”.

9. Line 181: Same question as the point 7. Please clarify the number of tardigrades.

REPLY: We changed the sentence to clarify the number of tuns. The change is as follows: “Ultrastructural analysis of tuns was performed using transmission electron microscopy (TEM). (The analysis was based on storage cells, which are regarded as useful for ultrastructure analysis aimed to estimate possibility of successful return of tuns to active life, due to the cells being described as a factor affecting survival rates during anhydrobiosis. Accordingly, the cells are described as the main form of energy storage, enabling a proper nutrient regime for different tissues as well as providing protection to tissues by producing protective metabolites. Moreover, it has been shown that energetic support is also important during the tun stage. Therefore, we assumed that tuns that were to survive should have had the correct ultrastructure of these cells, enabling their proper function also during/after rehydration.) Out of tuns obtained by protocols A and B, 10 tuns were randomly selected for each of the protocols.” (with relevant references). See also our answer to the comment 7. The reply also contains the answer to the comment 10, enclosed in parentheses.

10. Line 186: TEM ultrastructural analysis of specimen integrity is based only on storage cells according to a previous study in another species (Czernekova, 2018). Does the analysis of a single cell type provide sufficient information to perform the classification of degenerative states proposed in this manuscript?

REPLY: We decided to focus the TEM analysis on storage cells, because storage cells are described as a factor, affecting survival during anhydrobiosis (Jönsson and Rebecchi, 2002). These cells are regarded as the main form of energy storage, enabling a proper nutrient regime for different tissues as well as providing protection to tissues by producing protective metabolites (Reuner et al.,2010; Bertolani and Rebecchi, 2018; Schill and Hengherr, 2018). Moreover, it has been shown that energetic support is also important during the tun stage (Wojciechowska et al., 2021). Therefore, we assumed that tuns that were to survive should have had the correct ultrastructure of these cells enabling their proper function also during/after rehydration. Consequently, we stated that storage cells are regarded as useful cells for ultrastructure analysis aimed to estimate possibility of successful return of tuns to active life. It does not mean that we think that other cells should be excluded from the analysis. Furthermore, we agree that we should limit the observed state of degeneration only to storage cells and not apply the term to whole tuns.

Taking into account what was written before the following changes were introduced:

(1) the application of storage cells for TEM analysis was additionally explained to following: “The analysis was based on storage cells, which are regarded as useful for ultrastructure analysis aimed to estimate possibility of successful return of tuns to active life, due to the cells being described as a factor affecting survival rates during anhydrobiosis. Accordingly, the cells are described as the main form of energy storage, enabling a proper nutrient regime for different tissues as well as providing protection to tissues by producing protective metabolites. Moreover, it has been shown that energetic support is also important during the tun stage. Therefore, we assumed that tuns that were to survive should have had the correct ultrastructure of these cells, enabling their proper function also during/after rehydration.” (with relevant references).

(2) the term “degeneration” was limited to storage cells. This also applies to caption of Table 2.

11. Line 192: Authors describe mitochondria in Figure 3A. But the quality of the picture needs to be improved. This figure doesn’t allow to observe the state of the mitochondria or mitochondria cristae. We can’t conclude anything on mitochondria with this figure. The quality of Figure 3A needs to be improved to be interpreted with certainty.

REPLY: We added an additional image (part B) with higher magnification in order to make the ultrastructure of mitochondria more visible. Unfortunately, the ultrastructure of anhydrobiotic cells, including mitochondria is very difficult to be visualized by TEM. The same was stated for mitochondria by Richaud et al. (2020). The authors conclude that mitochondria in tuns appear to have shorter cristae of the inner mitochondrial membrane and because oxidative phosphorylation occurs mostly in the deeply invaginated cristae, it can be assumed that autophagy allows tardigrades to survive starvation. Therefore, we added the following sentence to the mentioned part of text: “As stated by Richaud et al. [47], mitochondria in tuns appear to have shorter cristae of the inner mitochondrial membrane and because oxidative phosphorylation occurs mostly in the deeply invaginated cristae it can be assumed that autophagy allows tardigrades to survive starvation. Accordingly, autophagosomes were observed sporadically in stage 0 (Fig. 3A).”

12. Line 194: Authors describe for Figure 3B: "single vacuole and autophagosomes appeared". But, according to me, we can also observe a vacuole in stage 0.

REPLY: In storage cells in stage 0 single autophagosomes can be observed sporadically (see Fig 3A) but we didn’t observe vacuoles in storage cells in stage 0. The information concerning autophagosomes in storage cells in stage 0 was added to the mentioned part of the text.

13. Line 198: A description of the mitochondria in Figure 3C is presented. However, the quality of the images does not allow for the degree of interpretation that is proposed. Like above, a better image quality is required to allow conclusions.

REPLY: We hope that all the details are more visible in the improved images. The Fig. 3C is now Fig. 3D

14. Lines 202 and 203: it is stated that "these observations corroborated with the survival rate" However, while the observations indicate 100% of storage cells degeneration one can also note that the corresponding mortality which is not 100% maintains a low survival rate. The analysis of only the storage cell type may not be discriminating enough to allow a totally reliable prediction in the absence of the analysis of at least one other cell type. Please adjust the conclusion of this section accordingly.

REPLY: The survival rate estimated after tun rehydration strongly correlates with the storage cell degeneration. Taking into account the functions of these cells, one could assume that the state of storage cells in tuns could be indicative of the tun survival. Nevertheless, ultrastructure analysis of other cell types would strengthen this conclusion. Therefore, the part was changed as follows: “Interestingly, these observations correlated with the survival rate determined for tuns obtained by A and B protocols (Fig. 2). Thus, it could be concluded that in the case of protocols A and B, tardigrades forming tuns without visible degeneration of storage cells appear to be able to successfully return to active life. Thus, it can be assumed that the state of storage cells in tuns could be indicative of successful anhydrobiosis. Nevertheless, ultrastructure analysis of other cell types would strengthen this conclusion, i.e. digestive cells, epidermal cells and oocytes.

15. Line 212: "we ca assume.." should be corrected to ""we can assume.."

REPLY: Corrected as indicated.

*Figures:*

Figure 1: The quality of the two tuns pictures is insufficient. Images are totally different and cannot be compared to each other. As described in the M&M section, two different stereomicroscopes are used. The exposure for the two pictures is also different. The picture for the protocol B doesn’t allow to correctly observe the tun. The resolution is not sufficient.

For the storage cells TEM pictures, we don’t have scale bar.

REPLY: As we wrote in the answers to the comments 5, 6 and 8, we changed the procedure of preparing images of tuns obtained by protocol B and corrected the Figure accordingly. We hope that tuns in the corrected images are now comparable.

Figure 1 shows the research scheme. Therefore, images obtained by TEM are small and have no scale bars. Figure 3 contains the bars as well as images of much better quality. We added the relevant information to the caption of Figure 1. The information is as follows: “High definition images obtained using TEM analysis, with relevant scale bars added, are presented in Figure 3”.

Figure 3: The quality of pictures needs to be improved. Status of mitochondria or cristae for example are not clearly distinguishable.

REPLY: We corrected the figure accordingly.

*Figures captions:*

Figure 1: A description of tuns in the middle of the figure is required. And it’s precisely pictures that cannot be compared.

REPLY: As we wrote in the answers to the comments 5, 6 and 8, we changed the procedure of preparing images of tuns obtained by protocol B and corrected the Figure accordingly. We hope that tuns in the corrected images are now comparable We have to mention that Figure 1 serves as a research scheme. Therefore, we added data on the size of both tuns to the relevant part of the text (the Result and Discussion section, line 186 in revised manuscript with track changes). So that the corrected part reads as follows: “As shown in the research scheme presented in Figure 1, protocols A and B led to contraction of the body and withdrawal of legs into the body cavity accompanied by loss of water from the body, resulting in a distinctly shrunken body shape and the body size between 100-130 µm. The latter is in the range of the body compaction observed for *Hys. exemplaris* tuns.” (with relevant reference).

Figure 2: Statistical t-test is necessary. The test is mentioned in the M&M section but we can’t find the result in the rest of the paper.

REPLY: We are sorry for the oversight. We added illustration of the t-test results to Fig. 2 and corrected the figure caption properly.

Figure 3: Scale bars are very different between pictures (0.38um, 0.53um, 0.42um,…). For an easy and good reading of images, it is necessary to homogenize the scale bars.

REPLY: We cannot homogenize the scale bars of the images because a greater magnification is needed to show some structures. The unification of the scale will result in the necessity to place very long bars in some images, which may obscure the analysed structures. In Fig. 3 parts, all bars have the same length (1 cm). This principle is often used in the imaging of cell ultrastructure.
